# Optimizing the Detection of Venous Invasion in Colorectal Cancer: The Ontario, Canada, Experience and Beyond

**DOI:** 10.3389/fonc.2014.00354

**Published:** 2015-01-05

**Authors:** Heather Dawson, Richard Kirsch, David K. Driman, David E. Messenger, Naziheh Assarzadegan, Robert H. Riddell

**Affiliations:** ^1^Department of Pathology and Laboratory Medicine, Mount Sinai Hospital and University of Toronto, Toronto, ON, Canada; ^2^Clinical Pathology Division, Institute of Pathology, University of Bern, Bern, Switzerland; ^3^Department of Pathology, London Health Sciences Centre, Western University, London, ON, Canada; ^4^Division of General Surgery, Taunton and Somerset NHS Foundation Trust, Taunton, UK

**Keywords:** venous invasion, colorectal cancer, elastin stain, prognostic marker, stage II colorectal cancer

## Abstract

Venous invasion (VI) is a well-established independent prognostic indicator in colorectal cancer (CRC). Its accurate detection is particularly important in stage II CRC as it may influence the decision to administer adjuvant therapy. The Royal College of Pathologists (RCPath) of the United Kingdom state that VI should be detected in at least 30% of CRC resection specimens. However, our experience in Ontario, Canada suggests that this (conservative) benchmark is rarely met. This article highlights the “Ontario experience” with respect to VI reporting and the key role that careful morphologic assessment, elastin staining and knowledge transfer has played in improving VI detection provincially and beyond.

## Introduction

Venous invasion (VI) is a well-established independent predictor of hematogenous spread and mortality in colorectal cancer (CRC) (1–8). Although the prognostic importance of VI has been recognized since the late 1930s (1, 9), its assessment remains one of the most poorly performed aspects of colorectal pathology reporting. Indeed it is surprising that many of the “giants” in the field that authored the early papers on colorectal carcinoma, and introduced the classification of colorectal carcinoma that forms the basis of the TNM classification, chose to use lymph node metastasis as a surrogate marker of the risk of metastatic disease rather than VI, a more direct measure. Unless tumor spreads via the lymphatics and thoracic duct, it needs to get into veins to disseminate, especially to the liver. Had they thought this way, the subject of this review may well have been completely obsolete, and the surprise (and power of “tradition”) is that it has taken us another 80 years to rethink what we do and how we do it, as reflected in this review.

Extramural venous invasion (EMVI) is a strong predictor of adverse outcome (4, 6, 10–14), while the prognostic significance of intramural venous invasion (IMVI) remains unclear. Several studies suggest that IMVI may impact outcome, albeit to a lesser degree than EMVI (6, 10, 15).

Venous invasion is of particular importance in stage II CRC as its detection may prompt oncologists to consider adjuvant chemotherapy. The importance of accurate risk stratification in stage II CRC will become increasingly relevant as the proportion of node-negative CRC is set to increase with the expansion of screening colonoscopy programs. Current evidence suggests that at least 70% of screening detected CRCs are node negative (16). Thus, the interest in measures other than nodal status to stratify risk of disease progression in CRC is likely to grow.

The importance of VI is recognized by the UK Royal College of Pathologists (RCPath), which has recently adjusted its minimum audit standard of VI detection to 30% in CRC resections (17). However, data from population-based studies indicate that this standard is rarely achieved (5, 8, 13, 18) with marked variability in the reported incidence of VI that ranges from 9 to 90% (8). Such variability is most likely to be attributed to differences in case mix, reporting criteria, sampling, use of special stains, and the diligence and skill of the reporting pathologist. Under-reporting of VI is particularly common outside of sub-specialist units (7, 10, 18, 19).

**Figure 1:**
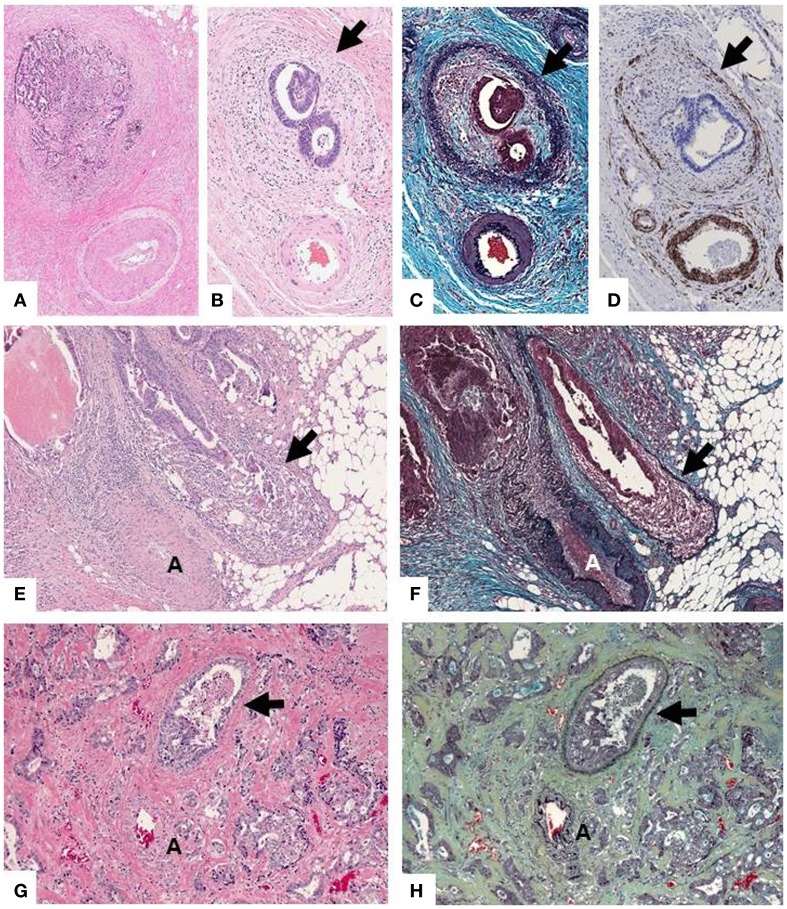
**(A,B)** “Orphan arteriole” sign (circumscribed tumor nodule adjacent to muscularized artery without an obvious accompanying vein). The residual vessel wall (arrow) can be highlighted with elastin trichrome stain **(C)** or immunohistochemical stain for caldesmon (smooth muscle marker) **(D)**, facilitating the detection of VI. **(E)** “Protruding tongue sign” (rounded tongue-like protrusion of tumor into pericolic fat adjacent to an artery [A]). **(F)** An elastin stain highlights elastin fibers of the residual vessel wall which has been partially obliterated by tumor. **(G)** In some instances detection of VI can be virtually impossible on (**H,E**) but easily recognized on the elastin stain **(H)**.

Accurate detection of VI can be challenging on routine H&E slides, especially when the muscular wall of the vein is obliterated beyond morphologic recognition or altered by pre-operative radiation (Figures 1G,H). In these circumstances, VI is easily overlooked unless key morphologic clues are sought. These include the so-called “orphan arteriole” sign (a circumscribed tumor nodule adjacent to muscularized artery without an obvious accompanying vein) (Figures 1A,B) and the “protruding tongue sign” (a smooth bordered protrusion of tumor into pericolic fat adjacent to an artery) (Figure 1E). Either of these findings, in the absence of a clearly visualized vessel wall, should prompt an elastin stain (Figures 1C,F) and/or a smooth muscle immunostain (Figure 1D), which will resolve the vast majority of equivocal cases (19, 20).

## The Role of Elastin Staining

There is a growing body of evidence that routine elastin staining substantially increases VI detection. Many studies have demonstrated the superiority of elastin staining compared to H&E alone in the assessment of VI, with the majority reporting a two to threefold increase in VI detection with elastin stains (15, 19, 21–24).

Furthermore, evidence suggests that VI assessed with an elastin stain may be a far better predictor of cancer survival than VI assessed by H&E alone. Roxburgh et al. (15) studied 419 patients undergoing curative CRC resection (before and after the introduction of routine elastin staining at their institution, *n* = 194 and 225, respectively) and found elastin-detected VI to be superior at predicting 3-year cancer-specific survival than VI detected by H&E alone. A follow-up study of 631 CRC resections found elastin-detected VI to be associated with a decrease in 5-year cancer-specific survival from 92 to 67% in node-negative disease (T1-4,N0) and from 79 to 42% in node positive disease (T1-4,N1/2). The combination of T-stage and elastin-detected VI was at least equivalent to T-stage and nodal status in predicting cancer-specific survival, and superior to TNM in node-negative disease (14). This led the authors to propose a “TVI” staging system as a simple alterative to TNM. Suzuki et al. (25), in a study of 124 patients with stage I CRC, found only elastin-detected VI to be an independent predictor of distant metastasis (VI detected on H&E alone was not significant on either univariate or multivariate analysis). Baumhoer et al. (26) failed to demonstrate a difference in 5-year survival between patients with and without elastin-detected VI in 185 patients with stage I and II CRC, but the study was limited by type II error due to the relatively small sample size.

Not surprisingly, the accuracy of VI detection on H&E has been shown to affect its prognostic power. Betge et al. (10) compared the prognostic significance of original vs. review diagnoses of VI (the latter by two experienced GI pathologists) in 381 CRC resection specimens. Review diagnoses of VI had a much stronger influence on both progression free survival [HR 3.97 (95% CI 2.71–5.81), *p* < 0.001 vs. HR 1.01 (95% CI 0.60–1.71), *p* = 0.96] and cancer-specific survival [HR 4.45 (95% CI 2.96–6.68), *p* < 0.001 vs. HR 1.05 (95% CI 0.60–1.84), *p* = 0.87] than did diagnoses made in the routine setting.

## The Ontario Experience

Institutional and provincial audits in the province of Ontario, Canada have revealed under-reporting of VI in CRC resections (18, 27) with a provincial VI detection rate of just 14% in 2010 (11% if academic hospitals were excluded)^1^. In order to determine the reasons for such under-reporting, a population-based survey of 361 Ontario pathologists was undertaken (18). A 15-item survey addressed reporting criteria (*n* = 6), the use of special stains (*n* = 5), and demographic information (*n* = 4). Pathologists were also asked to provide a self-estimate of their VI detection rates in CRC resection specimens (<10%; 10–19%; 20–29%; >30%). The overall response rate was 65%, which is relatively high for a survey of physicians. A majority of pathologists (70.2%) considered their VI detection rates to be <10%, with only 9% reporting VI detection rates >20%. Factors independently associated with estimated VI detection rates of ≥10% included (1) practice in a university-affiliated center, (2) a sub-specialist interest in GI pathology, and (3) application of the “orphan artery criterion” (i.e., tumor nodule adjacent to an artery where residual smooth muscle or elastin can be demonstrated on H&E or special stains) (18). Higher rates of VI detection among GI^1^ pathologists and those practicing in university-affiliated centers were also noted in institutional and provincial audits (18, 27) as well as in a study using a pre-defined set of cases (19). In the latter, the use of an elastin stain more than doubled VI detection by both GI and general pathologists (*p* = 0.001) and increased interobserver agreement for the detection of EMVI (H&E: κ = 0.23 vs. Elastin: κ = 0.41) (19).

The under-reporting of VI in Ontario, together with the heightened awareness generated by the population-based survey and the availability of a provincial mailing list presented a unique opportunity for practice improvement through knowledge transfer. Initially, an educational document was sent to all Ontario pathologists on the provincial mailing list providing the following information:

(1) Detailed feedback on the results of the VI survey
(2) Information regarding the prognostic importance of VI and the RC Path UK minimum standard for VI and
(3) A set of micrographs illustrating both the morphologic clues for VI detection (i.e., “orphan arteriole” and “protruding tongue” signs) and the utility of elastin staining in enhancing VI detection.

Broader national exposure was achieved by publication of a similar document in the Journal of Canadian Pathology (28). The impact of these initiatives is beginning to emerge at an institutional, provincial and national level. Many institutions in Ontario have since implemented routine elastin staining on all CRC resections. At our institution, elastin trichrome stains are performed on a minimum of five tumor-containing blocks per case. The associated costs are modest (about $3–4 per slide) and there has been no impact on turn-around time as stains are performed up front on pre-selected blocks. A recent audit of VI detection rates at Mount Sinai Hospital for the year prior to and the year following implementation of routine elastin staining revealed a twofold increase in VI detection rates (20.0–42.0%) that was independent of other clinicopathologic variables [adjusted Odds Ratio, 3.54 (95% confidence interval, 1.89–6.63); *p* < 0.001]. Comparable VI detection rates were achieved by both gastrointestinal (GI) and non-gastrointestinal (non-GI) pathologists (41 and 44%, respectively) (unpublished data).

Similar improvements in VI detection were observed among Ontario pathologists who had participated in the interobserver variability study VI in CRC (19, 29). Participants of this study were invited to submit pathology reports from all CRC resections issued in the 18 months prior to, and 18 months following completion of the study. Nine of the 12 pathologists (5 GI, 4 non-GI) submitted reports they had signed out (*n* = 233 pre-study and *n* = 216 post-study). Again, a two-fold increase in VI detection rates in the post-study period was noted [18.5–39.8% overall (*p* < 0.0001), 18.2–38.4% for GI pathologists (*p* < 0.001), and 19.1–45.6% for non-GI pathologists (*p* < 0.002)]. In addition, the mean number of elastin stains per case increased from 0.10 (SE, 0.41) to 2.51 (2.06) (*p* < 0.001), while the mean number of tumor-containing blocks per case remained constant [8.28 (6.69) vs. 8.12 (4.07); *p* = 0.761]. There were no significant differences in small vessel invasion, TNM stage, tumor location or proportion of patients receiving neoadjuvant therapy pre- and post-study.

The broader impact of knowledge transfer on VI detection at a provincial level was addressed by means of a follow-up survey of pathologists in Ontario. Participants were asked whether or not they considered their VI detection rates and use of elastin stains in CRC resections to have increased since the original 2010 VI practice pattern survey and receipt of the feedback/educational material that followed. Despite a relatively low response rate of 24%, 33 different hospitals were represented. Overall 67.5% of respondents considered their use of elastin stains to have increased, including 85% (11/13) of GI pathologists and 62% (38/61) of non-GI pathologists. Of those who reported increased use of elastin stains, 72% (36/50) felt that their VI detection rates had increased; this included 91% (10/11) of GI pathologists and 67% (26/39) of non-GI pathologists. Of those who reported no increase in elastin staining, only 8% (2/26) felt that their VI detection rates had increased. There was a highly significant association between the increased use of elastin staining and a perceived increase in VI detection (*p* < 0.0001). Finally, unpublished results from a survey of North American pathologists revealed that 46.5% of Canadian GI pathologists (20/43) routinely performed an elastin stain on at least one tumor-containing block on every CRC resection compared to less than 2% (1/58) of US GI pathologists. Differences were also seen between Canadian and US non-GI pathologists [19% (15/79) vs. 2% (3/139), respectively]. It would appear that knowledge transfer initiatives have influenced pathology practice with respect to VI reporting at both a provincial and national level in Canada.

## International Criteria for the Reporting of Vessel Invasion

Colorectal cancer reporting protocols of the RC Path (UK), Royal College of Pathologists of Australasia (RCPA), College of American Pathologists (CAP), and Japanese Society of Cancer of the Colon and Rectum (JSCCR) vary considerably with respect to blood vessel and lymphatic vessel invasion reporting.

The recently updated RC Path dataset (17) includes only venous (large vessel) invasion as a mandatory data element (reported as extramural, intramuscular or submucosal) and regards the evidence as insufficient for mandatory reporting of lymphatic and small vessel invasion. Talbot’s definition of VI as “tumor present in an endothelium-lined space surrounding a rim of smooth muscle or containing red blood cells” remains in use, but in addition the demonstration of a convincing elastic lamina around a tumor nodule is now considered sufficient to categorize as positive for VI, even if an endothelial-lined space is not visible. The guidelines recommend that VI is detected in at least 30% of CRC resections and that individual centers should monitor VI detection rates and consider routine elastin staining to facilitate its detection if the 30% minimum standard is not met (Table 1).

**Table 1:** **Recommendations for the detection of VI (8, 1,7)**.

A minimum of four or five tumor blocks [as included in most sampling protocols (17)] should be submitted for optimum assessment of VI.
When submitting blocks, areas of linear spiculation at the infiltrating edge of the tumor should be targeted for histological examination.
In rectal cancers, detection of VI should be guided by MRI findings in terms of the presence or absence of EMVI. If EMVI is reported present on imaging, every effort should be made to detect EMVI including additional sampling, careful histologic examination and elastin stains (if not already implemented).
Morphological clues play a key role in detecting VI. Particular attention should be paid to the presence of the orphan arteriole or protruding tongue signs on H&E. Elastin stains should be performed on all blocks equivocal for VI.
Individual departments should monitor their VI detection rates with regard to the UK RC Path minimum audit standard of 30% in all CRC resections.
In departments where this benchmark is not met, routine elastin staining of most or all tumor blocks should be considered. When ordered at the time of grossing, this is associated with only minimal additional costs, and there is no significant increase in turn-around times.

The RCPA guidelines (30) recognize the significance of both large vessel (venous) and small vessel (lymphatic and capillary) invasion and recommend separate documentation of each as well as the distinction between EMVI and IMVI. The use of histochemical and immunohistochemical stains is encouraged if there is a suspicion of VI on H&E.

While the CAP reporting guidelines (31) recognize the superior prognostic significance of EMVI, all forms of blood vessel and lymphatic invasion (large and small vessel) are grouped under the broad term “lymph-vascular invasion.” Currently there are no recommendations regarding the use of special stains to identify blood vessel or lymphatic invasion.

Consensus guidelines for the assessment of lymphatic and blood vessel invasion from the Pathology Working Group of the JSCCR have recently been developed using the Delphi method (32). Diagnostic criteria for elastica-detected VI and D2-40-detected LI include an elastic membrane covering more than half of the circumference of a tumor cluster even without an accompanying artery or vessel structure and D2-40 positive endothelial cells covering at least half of the circumference of a tumor cluster, respectively. However, a recent international study group found that attempts to apply these criteria were associated with a decrease in interobserver agreement (33). JSCCR guidelines suggest the separate reporting of blood vessel and lymphatic vessel invasion, but do not distinguish the size of the vessel.

The variation in national/regional pathology reporting guidelines for blood and lymphatic vessel invasion may influence patient management. While most national guidelines recommend that adjuvant chemotherapy be offered in “high-risk” stage II disease (34, 35), precisely what defines “high-risk” in terms of vessel invasion differs regionally. For instance, the US-based National Comprehensive Cancer Network considers any type of blood or lymphatic vessel invasion (34) as a high-risk feature in Stage II CRC; this is in line with the CAP guidelines grouping of all forms of blood and lymphatic vessel invasion in single category (“lymph-vascular invasion”). In contrast, EMVI but not other types of blood/lymphatic vessel invasion are considered “high-risk” by the National Institute for Health and Care Excellence in the UK (35) in line with UK pathology guidelines requiring separate reporting of EMVI and which do not include small vessel invasion as a mandatory data element.

Finally, while current guidelines recommend chemotherapy be offered in stage II CRC with any “high-risk” feature, the relative benefit of chemotherapy for individual “high-risk” features (such as VI) remains to be determined. Attempts to determine the relative benefit of chemotherapy for VI in stage II CRC are currently limited by (1) low VI detection rates and lack of central review in some major trials [e.g., VI rate of 13% in the QUASAR (36) and 8.9% of Stage II patients in the MOSAIC trial (37)], (2) insensitive methods for VI detection (i.e., H&E stain alone), and (3) relatively small numbers when subgroup analyses are performed. Therefore prospective studies to determine the relative benefit of chemotherapy in patients with elastin-detected VI would be of considerable interest and importance.

## Conclusion

Venous invasion, particularly when in an extramural location, is a powerful prognostic factor in CRC. Its presence in stage II CRC may prompt oncologists to offer adjuvant chemotherapy. Institutional and provincial audits in Ontario identified widespread under-reporting of VI, which led to several knowledge transfer initiatives in the province. These appear to have had an impact on the detection and reporting of VI both at a provincial and national level. In particular, recognition of key diagnostic clues, such as the “orphan artery sign” and “protruding tongue sign” and use of elastin staining can substantially increase VI detection rates. In turn, the more accurate/sensitive detection of VI has been shown to increase its prognostic power. Future challenges include establishing evidence based internationally accepted guidelines for the definition and reporting of blood vessel and lymphatic invasion, and implementing of methods to improve their detection.

## Conflict of Interest Statement

The authors declare that the research was conducted in the absence of any commercial or financial relationships that could be construed as a potential conflict of interest.

## Footnotes

^1^During this period, provincial reporting was performed according to the College of American Pathologists checklist (version 6) based on AJCC/UICC TNM 6th edition which required separate reporting of large vessel (venous) and small vessel invasion.
